# Lipoperoxide Nanoemulsion as Adjuvant in Cisplatin Cancer Therapy: In Vitro Study on Human Colon Adenocarcinoma DLD-1 Cells

**DOI:** 10.3390/nano11061365

**Published:** 2021-05-21

**Authors:** Stefania Vernazza, Elena Dellacasa, Sara Tirendi, Laura Pastorino, Anna Maria Bassi

**Affiliations:** 1 Department of Experimental Medicine, University of Genoa, 16132 Genoa, Italy; tirendisara@gmail.com (S.T.); Anna.Maria.Bassi@unige.it (A.M.B.); 2 Department of Informatics, Bioengineering, Robotics and Systems Engineering, University of Genoa, 16145 Genoa, Italy; elena.dellacasa@edu.unige.it (E.D.); laura.pastorino@unige.it (L.P.); 3 Inter-University Center for the Promotion of the 3Rs Principles in Teaching & Research (Centro 3R), 56122 Pisa, Italy

**Keywords:** invitro study, cancer cells, nanoemulsion, cisplatin, anticancer effects

## Abstract

Cisplatin is a first-choice chemotherapeutic agent used to treat solid tumors even though the onset of multi-drug resistance and the time–dose side-effects impair its mono-therapeutic application. Therefore, new drug-delivery approaches, based on nanomedicine strategies, are needed to enhance its therapeutic potential in favor of a dose-reduction of cisplatin. Polyunsaturated fatty acids and their metabolism-derived intermediates, as well as lipid peroxidation end-products, are used as adjuvants to improve the effectiveness of chemotherapy. Lipid hydroperoxides, derived from the oxidation of edible oils, can contribute to cell death, generating breakdown products (e.g., reactive aldehydes). In this regard, the aim of this present study was to evaluate an invitro combinatory strategy between a lecithin-based nanoemulsion system of K600, a patented mixture of peroxidated oil and peroxidated cholesterol, and cisplatin on DLD1 human adenocarcinoma cells. Our findings showed that nanoemulsions, acting in synergy with cisplatin, improve cisplatin bioactivity, in terms of enhancing its anti-cancer activity, towards DLD1 cells. Indeed, this combination approach, whilst maintaining cisplatin at low concentrations, induces a significant reduction in DLD1 cell viability, an increase in pro-apoptotic markers, and genotoxic damage. Therefore, K600 nanoemulsions as an efficient targeted delivery system of cisplatin allow for the reduction in the chemotherapeutic agent doses.

## 1. Introduction

The success of chemotherapy is restricted by multi-drug resistance (MDR) within cancer cells. The genomic instability of cancer cells may increase their survivability due to adaptive molecular mechanisms in response to the antiblastic drug used. Therefore, it is often necessary to carry out a combination of multi-chemotherapeutic agents, multi-drug therapy or even suspend therapy in order to reach a positive prognosis for the patient. As known, cisplatin is still a first-choice chemotherapy for several cancer treatments despite the onset of MDR and time–dose side-effects impairing its monotherapeutic application [1,2,3,4,5]. In this regard, several previous studies have shown that, to reach a better therapeutic efficacy, it is necessary to exploit the synergy between drugs and to develop new drug-delivery approaches based on nanomedicine [6,7].

It is well-known that most chemotherapeutic agents through redox regulation enhance the intracellular oxidative stress rate, leading to cancer cell death (i.e., arsenic trioxide, doxorubicin, and PEITC) [8,9,10,11].

From this perspective, the ‘oxidation therapy’, based on PUFAs [12,13], or PUFA’s metabolically derived intermediates or lipid peroxidation end-products [14,15,16], plays an adjuvant role in the effectiveness of chemotherapy. In particular, lipid hydroperoxides (LOOHs), intermediates of the metabolism of edible oil oxidation, are considered the mediators of cell death, resulting in the generation of breakdown products (i.e., reactive aldehydes) [17].

In this regard, our study assessed, using an invitro model, the combinatory strategy between nanoemulsions of K600, a patented peroxidated oil and peroxidated cholesterol-based mixture, rich in LOOHs, (the so-called NK600), and cisplatin (CCDP)on a human adenocarcinoma cell line (DLD1), according to oxidation therapy and nanomedicine approaches.

The nanoemulsion form can, indeed, improve the solubilization of low water-soluble chemotherapeutic drugs and their bioavailability because, thanks to their small size, they can easily penetrate cell membranes without damaging them [18,19,20].

## 2. Material and Methods

### 2.1. Cell Cultures

Human colon cancer cell lines, DLD-1, were obtained from The Biological Bank and Cell Factory (IRCCS Ospedale Policlinico San Martino–IST Istituto Nazionale per la Ricerca sul Cancro) and were maintained in DMEM (Dulbecco’s modified Eagle’s medium- Euroclone^®^, Milan, Italy) supplemented with 10% FBS and2 mM of glutamine (Euroclone^®^ Milan, Italy), w/o antibiotics.

DLD-1 were cultured at 37 °C in a humidified incubator containing 5% CO_2_. Cells were subcultured by TripLE™ Express (Invitrogen Life Technologies, Carlsbad, CA, USA) treatment when the original flask was approximately 75% confluent. All cell cultures were found to be mycoplasma-free during regular checks with the Reagent Set Mycoplasma Euroclone (Euroclone^®^ Milan, Italy).

### 2.2. NK600 Preparations

Extra-virgin olive oil (free of heavy metals) was heated to 80 °C and was added to 10% of certified heavy-metal-free cholesterol. The resulting mixture was then exposed to a UV source reaching a peroxide level of 600 EqO_2_/kg (see Supplementary Materials. European Patent Specification, international publication number: WO 2009060493 A1).

The emulsifying solution was prepared by dissolving 1 wt.% of commercial granular soybean lecithin in 0.01 M of PBS at pH 7.4 (Sigma-Aldrich, Darmstadt, Germany ) with continuous stirring for 1 h. Then, 5 wt.% K600 oil was added to the solution and was vortexed for 5 min. The samples, 1 mL each, were treated by batch ultrasonic homogenization at 40 kHz for 1 min. The obtained emulsion was stored in the dark at room temperature. For invitro testing, the emulsions were diluted in DMEM and were passed through 0.22 μm syringe filters.

### 2.3. N-K600 Size and Electrophoretic Measurements

The mean particle size and electrophoretic mobility (zeta potential, ζ) of the nanoemulsion were determined by dynamic laser light scattering (DLS) using a Malvern Zeta sizer Nano ZS (Malvern Instruments, Worcestershire, UK). The samples were diluted to a droplet concentration of 0.5 wt.% with buffer solution. A refractive index of 1.467 was used to calculate the particle size distribution. All samples were measured in triplicate and measurements were performed in five repetitions.

### 2.4. N-K600 Stability

Samples were stored at room temperature in the dark for up to one month. The mean particle size and electrophoretic mobility were analyzed during the storage time, as described above.

### 2.5. Cisplatin-CCDP

Cisplatin powder (Cisplatin CRS, European Pharmacopoeia Reference Standard) was dissolved in 0.9% sodium chloride solution and was filtered with 0.22 μm syringe filters, reaching a stock solution final concentration of 1mg/mL.

### 2.6. Experimental Conditions

The effects of NK600, CCDP, and several combinations (Combos) of these 2 compounds (NK600 + CCDP) on DLD1 were carried out up to a maximum of 48 h of exposure.

Both the single compounds and the Combos, in terms of concentrations and exposure times, differed according to the specific test performed (Table 1).

### 2.7. MTT Assay

At the end of each experimental treatment, the cell viability, in terms of mitochondria functionality, was assessed in DLD1 via MTT assay. The optical densities (ODs) of the dissolved formazan crystals were determined spectrophotometrically at 570 nm. The quantification of cell viability was obtained by comparing the optical density of the extracts, and the relative cell viability was calculated for each tissue as a percentage of the mean of the negative control tissues [21].

In order to evaluate if the NK600 and CCDP combination, compared to the single compounds, achieved an improved therapeutic result on DLD-1, the data were analyzed by CompuSyn software (ComboSyn, Inc., Paramus, NJ. USA). The combination index (CI), obtained from the several combinations, allowed us to study the possible biological/metabolism interactions between the drugs or compounds in order to evaluate the antagonism (CI > 1), additive (C = 1), or synergistic effects (C < 1) [22,23].

### 2.8. DNA Assay

The cellular DNA content for cell proliferation was measured by the method of Rao and Otto (1992) [24]. At each check point time, the experimental medium was removed and, after washing with warm PBS, 1 mL of lysis solution (urea 10 M, 0.01% SDS in saline sodium citrate buffer [SSC], 0.154 M of NaCl, 0.015 M of Na3 citrate, pH 7) was added to each well. The dissolved cell suspensions were incubated at 37 °C in a shaking bath for 2 h, and then 1 mL of Hoechst 33258 dye, 1 mg/mL in SSC buffer, was added in the dark. The absorbance was measured with an LS5 Perkin Elmer spectrofluorometer at excitation and emission wavelengths of 355 and 460 nm, respectively. The cell proliferation was estimated by referring fluorescence units to a linear standard curve for DNA fluorescence versus cell number.

### 2.9. qPCR

The total RNA was extracted using the QuickRNA^TM^MiniPrepKit (Zymo Research, Irvine, CA, USA) according to the manufacturer’s instructions. The quality and quantity of RNA were analyzed using a NanoDrop spectrophotometer (Nanodrop Technologies, Wilmington, DE, USA). The cDNA was synthesized by using a SuperScript^TM^ III First Strand Synthesis System (ThermoFisher Scientific, Milan, Italy). The Primer Assay—Plate (Bio-Rad, Milan, Italy) was used for PCR reaction, according to the manufacturer’s instructions. All samples were analyzed in triplicate (Table 2).

### 2.10. Western Blotting

#### 2.10.1. Nuclear/Cytosol Fractionation

The nuclear/cytosol fractionation on DLD-1 was performed using a lysis buffer solution with HEPES, 2 mM of MgCl_2_, 15 mM of KCl, 0.1 mM of EDTA, 1% 0.1 M of 1,4-dithiothreitol, and 1% protease inhibitor-Complete^TM^ ULTRA tablets (Merck KGaA, Darmstadt, Germany). After 20 min at 4°C, for each sample,0.05% (v/v) Nonidet^TM^-P40(Merck KGaA) was added. Then, the suspension was centrifuged at 10,000×*g* to split the nuclear fraction from the cytosol fraction. The nuclear pellet was resuspended in a volume of HEPES, 300 mM of NaCl, 50 mM of KCl, 0.1 mM of EDTA, 10% glycerol, 1% 0.1 M of DTT, and 1% protease inhibitor-Complete^TM^ ULTRA tablets (Merck KGaA) lysis buffer, and it was sonicated and incubated ON at 4 °C on a rotary shaker. The cytosol fraction was stored at −80 °C. The following day, each sample containing the nuclear fraction was centrifuged at 10,000 × *g* and the supernatant was stored at −80 °C.

#### 2.10.2. Whole Lysates collection

Cell lysates were collected using RIPA buffer (Sigma Aldrich, Darmstadt, Germany) plus a protease inhibitor cocktail (Complete Tablets, Roche Diagnostic GmbH, Mannheim, Germany), and they were sonicated until solubilized.

#### 2.10.3. Immunoblotting

Protein measurement was carried out using a Pierce^TM^BCA Protein assay Kit (Thermo Scientific, Rockford, IL, USA). An equal amount of protein was resolved in Ani kD^TM^ mini precast gel (Bio-Rad Laboratories, Inc., Hercules, CA, USA) in anSDS-PAGE Running Buffer, and it was transferred onto a PVDF membrane (Thermo Scientific, Rockford, IL, USA) and probed with primary antibodies (Table 3), followed by incubation with HRP-conjugated secondary antibodies (NA9340V and NA931V, against rabbit and mouse primary antibodies, respectively, Amersham Life Science, Milano, Italy). The proteins were detected by Western Bright^TM^ ECL (Advansta, CA, USA), exposed to film, and analyzed using a BIORAD Geldoc 2000. The data presented were calculated after normalization with GAPDH/HADAC1. Densitometrical data obtained from Quantity One software (Bio-Rad) were applied for statistical analysis and normalized against the housekeeping GAPDH/HADAC1.

### 2.11. GSH Measurement

Reduced glutathione (GSH) was determined in DLD1 according to the procedure described by Hissin and Hilf [25]. The cells (5 × 10^6^ cells/mL), washed out from incubation medium, were treated with 5% TCA solution, as a protein precipitant. Upon centrifugation, the GSH content in the supernatant was determined by reaction with o-phthaldialdehyde (OPT), and the resulting fluorescence was monitored [26].

### 2.12. Statistical Analysis

The data are represented as means ± standard deviation of the mean of three independent experiments performed in triplicate. The significance was assessed by one-way analysis of ANOVA variance followed by both posttests: Dunnett’s test vs. untreated DLD-1 cultures, and Bonferroni’s test for multiple comparison, using GraphPad Prism for Windows (version 5.03 and GraphPad Software, Inc., La Jolla, CA, USA). Statistically significant differences were set at *p* < 0.05 and *p* < 0.01.

## 3. Results

### 3.1. NK600 Characterization

**Figure 1:**
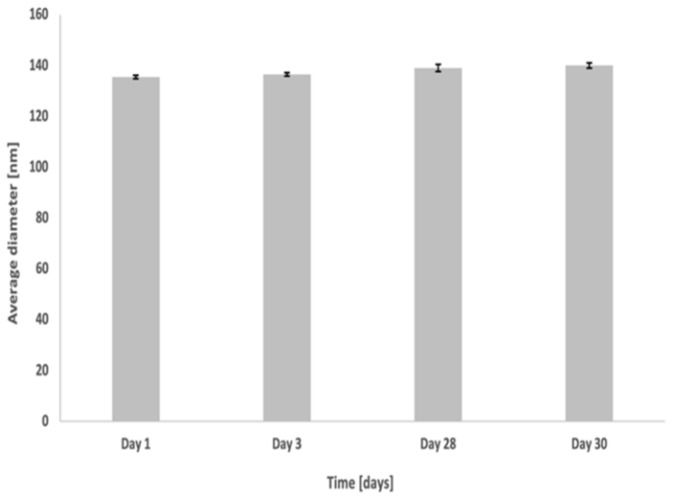
Stability of nanoemulsion droplets size over time.

The nanoemulsion droplets were characterized at room temperature, immediately after their preparation. The average zeta potential was found to be −49.53 ± 2.30 mV, indicating a strong repulsive force among the droplets, therefore suggesting that the nanoemulsion was physically stable [27]. The average diameter of the particles was found to be 140 ± 2 nm, with a polydispersity index (PI) of 0.226, indicating a highly uniform distribution [28]. In order to assess the stability of nanoemulsion over time, the average diameter was monitored for up to one month (Figure 1). Nanoemulsions showed high stability in terms of both the average diameter and the zeta potential throughout the storage period.

### 3.2. Synergic Effect of NK600-CCDP Combination

**Figure 2:**
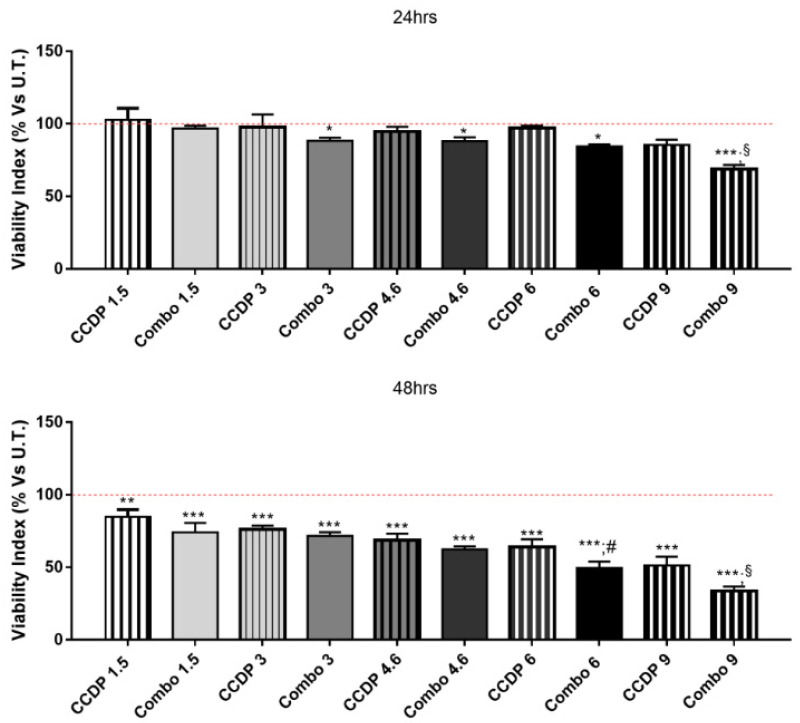
DLD-1 viability index. Viability indices of untreated and treated DLD-1 wereanalyzed viaMTT assay after 24 and 48 h. The data are expressed as percentages vs. viability index of untreated DLD-1 (represented in the graphs as the red dotted line), and each value represents the mean ± SD of 3 separate experiments running in triplicate. The CCDP concentrations are expressed as µg/mL; Combo units are in µg/mL of CCDP and mg/mL of NK600 in each Combo.*** *p* < 0.001; ** *p* < 0.01; * *p* < 0.05 vs. untreated DLD1; ^§^ *p* < 0.001; ^#^ *p* < 0.01 vs. CCDP alone, untreated DLD1 (one-way ANOVA).

In order to investigate the effects of the association between NK600 and CCDP (Combo),we analyzed the viability index of the DLD-1 human adenocarcinoma cell line via MTT assay at different time points, from 24 to 48h. DLD1 cells were treated with increasing concentrations of CCDP alone, ranging from 1.5 to 9µg/mL, and with the Combos (1.5–9µg/mL of CCDP+ and 1.5–9 mg/mL of NK600). As shown in Figure 2, the viability of DLD-1 cells was more compromised by the Combo treatment compared to CCDP in a concentration-dependent manner.

**Figure 3:**
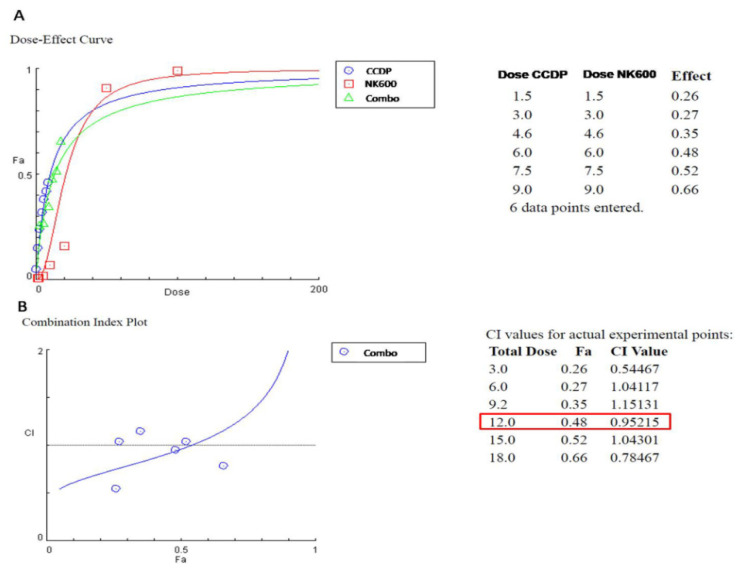
Compusynanalysis. (**A**)The dose–effect analysis of both single compounds and their combination was determined by Compusyn software, and the results are shown in the sigmoid concentration–effect curves. (**B**)The combination index (CI) values were determined by Compusyn software for all of the combined treatments.

To determine which of the Combos have reduced cell viability due to a synergic effect, we used Compusyn software, a computerized quantitation of synergism and antagonism (Figure 3). Indeed, it is of interest to understand whether the use of multiple compounds may have the same target or several targets in order to identify a more effective treatment for colon adenocarcinoma cells. This test, as a synergism outcome, showed that only some tested ‘Combos’ were able to increase the efficacy of the therapeutic effects, namely Combo 1.5, Combo 6, and Combo 9. Our studies then focused on testing just Combo 6 (CCDP 6 µg/mL and NK600 6 mg/mL) because it highlighted both a Fraction affected (Fa) of about 0.48 and a combination index (CI) of 0.95, which specify, respectively, half of the maximal inhibitory concentration (IC50) and the synergic effect of such a Combo.

### 3.3. Basal Nuclear Levels of NRF2 and Gene/Protein Involved in the NRF2 Pathway on DLD1 Cells Treated with CCDP, NK600, and Combo 6

**Figure 4:**
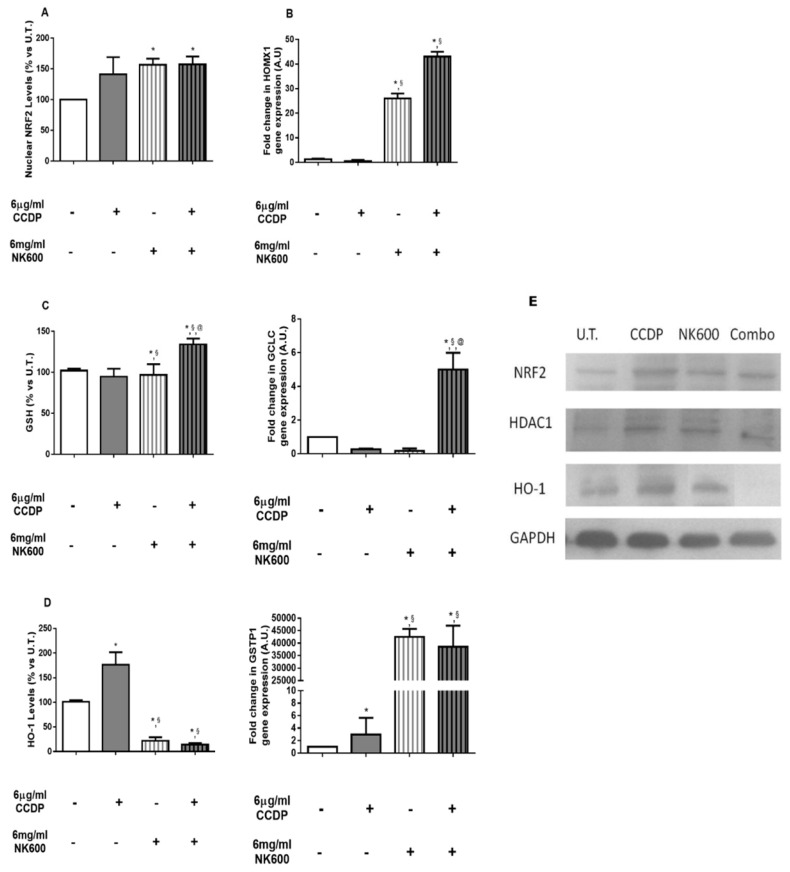
Antioxidant response analysis. (**A**) Nuclear levels of NRF2 protein were evaluated in DLD1 after 6 h of 6 µg/mL of CCDP, 6 mg/mL of NK600, and Combo 6 exposure. The analysis was performed by immunoblotting and the bars are expressed as % vs. untreated (U.T.) DLD1 cultures. (**B**) Gene expression analysis was performed on DLD1 after 12 h of 6 µg/mL of CCDP, 6 mg/mL of NK600, and Combo 6 exposure.HOMOX1, GCLC, and GSTP1 data are expressed as fold-increases relative to untreated cultures at the same end-point, and they are normalized to GAPDH housekeeping gene expression. (**C**) GSH levels in DLD1 after 24 h of 6 µg/mL of CCDP, 6 mg/mL of NK600, and Combo 6 exposure. The cellular GSH concentration was determined by fluorimetric analysis and is expressed as % vs. U.T. cells. (**D**) HO-1 protein levels were evaluated in DLD1 after 48 h of 6 µg/mL of CCDP, 6 mg/mL of NK600, and Combo 6 exposure. The analysis was performed by immunoblotting and the bars are expressed as % vs. untreated (U.T.) DLD1 cultures. (**E**) The figures depicted are representative of at least three similar immunoblot analyses: NRF2 and HO-1 protein levels in untreated DLD1, and in treated DLD1 (6 µg/mL of CCDP, 6 mg/mL of NK600, and Combo 6). HDAC1 and GAPDH were used as internal controls for equal protein loading on gels. The data represent the mean ± standard deviation (SD) of 3 independent experiments. * treated DLD1 vs. U.T. DLD1; §CCDP vs. NK600/Combo6; @NK600 vs. CCDP/Combo 6. * *p* < 0.01; ^§^ *p* < 0.01; ^@^ *p* < 0.01 (two-way ANOVA followed by Bonferroni’s test).

Nuclear NRF2 levels analyzed on nuclear fractions of DLD1 cells, using Western blotting analysis, were seen to increase at 6 h with this ranking: CCDP 6 µg/mL > NK600 6 mg/mL > Combo 6 (Figure 4A). NRF2 is a transcription factor and its translocation from the cytoplasm into the nucleus can increase the antioxidant gene transcription [29,30]. Next, we determined the expression levels of representative genes involved in the NRF2 pathway, normalized vs. GAPDH gene expression levels by qRT-PCR. Indeed, the HOMOX1 and GCLC levels at 12 h showed an increased capacity for an antioxidant defense by Combo 6, whereas GSTP1 levels were also increased by NK600 6 mg/mL (Figure 4B). Therefore, we assumed that the ROS imbalance, induced by Combo 6 on DLD1, was stronger than CCDP and that it may be a key modulator of NRF2 activity. In fact, after 24 h of experimental procedures, we observed a significant increase in GSH levels in DLD1 treated with NK600, as well as with Combo 6 (Figure 4C). As known, GSH, being the most important antioxidant element in mammalian cells, is often responsible for reducing the intracellular accumulation of CCDP [31,32,33,34,35]. However, to confirm whether or not Combo-induced GSH levels were able to modulate the CCDP sensitivity of DLD1, we analyzed the HO-1 protein levels at 48 h (Figure 4D). Surprisingly, the reduction in the HO-1 protein after Combo 6 and NK600, but not after CCDP, suggested that DLD1 cells lose protection over time against stress conditions, thus increasing the level of cell death [30,36,37].

### 3.4. P53-Dependent Pathway Interferes with Antioxidant Responses

**Figure 5:**
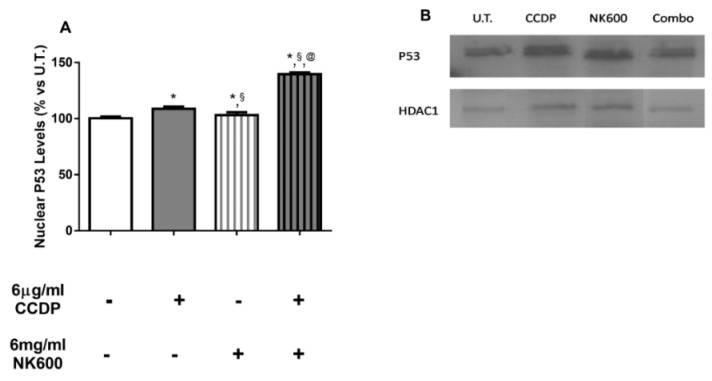
Apoptosis analysis (immunoblotting). (**A**) Nuclear levels of p53 protein were evaluated in DLD1 after 3 h of 6 µg/mL of CCDP, 6 mg/mL of NK600, and Combo 6 exposure. The bars are expressed as % vs. untreated (U.T.) DLD1 cultures. (**B**) The figures depicted are representative of at least three similar immunoblot analysis p53 protein levels in untreated DLD1, and in treated DLD1 (6 µg/mL of CCDP, 6 mg/mL of NK600, and Combo 6). HDAC1 was used as internal controls for equal protein loading on gels. The data represent the mean ± standard deviation (SD) of 3 independent experiments. * treated DLD1 vs. U.T. DLD1; §CCDP vs. NK600/Combo6; @NK600 vs. CCDP/Combo 6. * *p* < 0.01; ^§^ *p* < 0.01; ^@^ *p* < 0.01 (two-way ANOVA followed by Bonferroni’s test).

P53 is a transcription factor with a central role in genome integrity maintenance. As known, under stress conditions, the p53-pathway can modulate several proteins and microRNAs involved in cell cycle arrest, DNA repair or apoptosis, inhibiting tumor development [38,39]. Unfortunately, its protective role is defective in several cancers. Therefore, in order to understand the p53 role in DLD1 cells, we first evaluated its nuclear levels, using Western blot analysis, at 3 h of experimental treatment. These results revealed an increasedp53 nuclear translocation by 6 µg/mL of CCDP and markedly by Combo 6 (Figure 5A,B).

**Figure 6:**
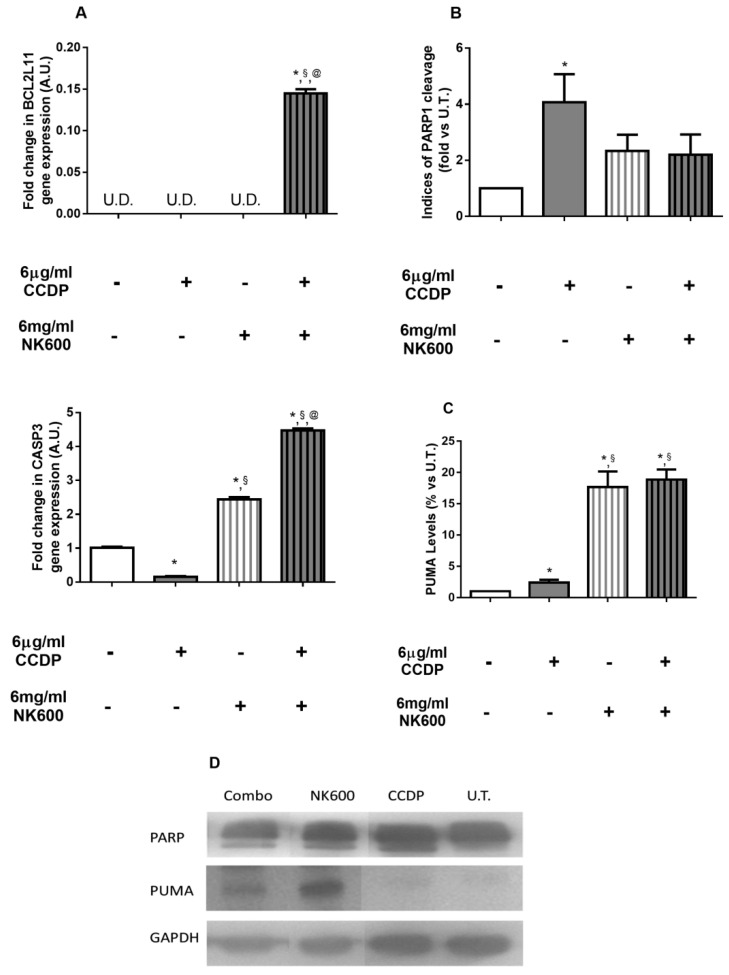
Apoptosis analysis. (**A**) Gene expression analysis was performed on DLD1 after 12 h of 6 µg/mLof CCDP, 6 mg/mLof NK600, and Combo 6 exposure.BCL2L11 and CASP3. Data are expressed as fold-increases relative to untreated cultures at the same end-point and are normalized to the GAPDH housekeeping gene expression. (**B**,**C**) Immunoblotting analysis. Bars represent the ratio of cleaved PARP(**B**) and Puma protein levels (**C**), and the data are expressed as fold values vs. U.T. DLD1 cells. (**D**) The figures depicted are representative of at least three similar immunoblot analyses of PARP and PUMA protein levels in untreated DLD1, and in treated DLD1 (6 µg/mL of CCDP, 6 mg/mLof NK600, and Combo 6). GAPDH was used as an internal control for equal protein loading on gel. The data represent the mean ± standard deviation (SD) of 3 independent experiments. * treated DLD1 vs. U.T. DLD1; §CCDP vs. NK600/Combo6; @NK600 vs. CCDP/Combo 6. * *p* < 0.01; ^§^ *p* < 0.01; ^@^ *p*< 0.01 (two-way ANOVA followed by Bonferroni’s test).

The qRT-PCR analysis detected that the BCL2L11 expression, the Bim protein gene, was increased by Combo 6 solely at 12 h, confirming its greater ability to trigger apoptosis (Figure 6A). Furthermore, to support the apoptosis hypothesis, we assessed the PUMA protein levels and PARP cleavage fragments using Western blot analysis (Figure 6B–D). PUMA belongs to ’BH3-only’ and it is an essential apoptosis p53-mediator [40,41,42,43,44,45,46]. This obtained result confirmed the apoptotic role of Combo 6 compared to CCDP. Indeed, the addition of NK600 to CCDP (Combo 6) greatly increased the PUMA expression (Figure 6C,D). Finally, we evaluated the CASP3 gene expression (Figure 6A) and one of its protein targets, the PARP cleavage (Figure 6B,D), using qRT-PCR and Western blot analysis, respectively [47,48,49]. In this case, at 6 h, the CASP3 gene amplification resulted in being more increased by Combo 6 whilst being decreased by CCDP treatment alone. However, although the PARP cleavage index showed that both Combo 6 and 6 µg/mL of CCDP were able to trigger DLD1 cell apoptosis, it was observed that CCDP alone induced the stronger effect of the two.

## 4. Discussion and Conclusions

Some of the traditional cancer therapy approaches, including chemotherapy, surgery and radiotherapy, today still show limited efficacy due to their side effects [50]. Moreover, when such traditional approaches are used as a single modality, they do not necessarily provide satisfactory outcomes, as in the case of surgery, where it is not always possible to carry out a complete tumor lesion resection [51], or in the case of chemotherapy and radiotherapy, where there is the onset of both MDR [52,53] and the insensitivity of hypoxic cancer cells to ionizing radiation [54,55].

In this regard, several past studies have demonstrated that the effects of certain treatments can be enhanced if used in combination with other treatments, resulting in considering the combination therapy approach as a valid alternative to the monotherapeutic approach [56,57,58,59]. In fact, in clinical cancer treatments, the combined therapy, that is, either of a co-delivery of two or more therapeutic agents or of a combination of different treatments, shows a reduced individual drug-related toxicity and suppressed MDR in addition to synergistic anti-cancer effects.

This present work aims to demonstrate that the CCDP effects can be enhanced by the combinatory use of K600 nanoemulsion in vitro on the DLD1human adenocarcinoma cell line. Indeed, the synergistic effects of some Combos (CCPD/NK600 combinations) proved their ability to reduce DLD1 viability in a dose-dependent manner compared to CCDP alone at the same dose (Figure 2 and Figure 3). However, from the analysis of the results obtained with the various Combos, Combo 6 was chosen because it exhibited a more potent effect (IC50 = 6 µg/mL CCPD + 6 mg/mL NK600) than the others in DLD1 cells.

In order to verify whether the reduction in cell viability was due to an increase of oxidative stress rate so much so as to trigger apoptosis, we analyzed both the antioxidant response and the apoptotic response.

As known, under oxidative stress insult, cells mainly activate the p53 and NRF2 pathways in order to enhance their ability to mitigate such conditions. Indeed, several findings have supported the existence of a p53-NRF2 cross-talk through a positive feedback loop [60,61], which becomes crucial in mutant p53cancer cells due to them gaining an improved survival to oxidative stress [62]. Moreover, in several cancers, somatic mutations, either in NRF2 or in KEAP1, prevent NRF2 degradation, often resulting in a poor prognosis given that the high NRF2 expression or its nuclear localization is correlated with reduced oxidative stress and an increased MDR [63].

In our cancer cell model, untreated DLD1 cells showed lower basal levels of nuclear NRF2 compared to those treated with either Combo 6 or single treatments (6 µg/mL of CCDP or 6 mg/mL of NK600) even though a significant increase (*p* < 0.01) of both the selected NRF2 downstream gene expressions analyzed and the GSH levels were observed only in the Combo 6-treated DLD1 (Figure 4).

However, Combo 6 treatment and, to a lesser extent, NK600 alone, showed to activate the apoptotic pathway, as evidenced by the increase in PUMA protein levels, as well as PARP cleavage index (Figure 5 and Figure 6), and so, we have speculated that the combination of CCDP and NK600 was able to target the anti-oxidative stress modules, thus increasing the cellular ROS and, consequently, promoting cell death.

Although we do not know the actual mechanism by which Combo 6 is able to sensitize DLD1 to apoptosis, in this study, we have demonstrated that such a combination therapy enhances the efficacy of CCDP in favor of its dose-reduction. Therefore, it can be considered as a preliminary approach towards a combination therapy of lipo-peroxide nanoemulsion (NK600) as a pro-oxidant agent with a traditional chemotherapeutic compound. However, further studies using other chemotherapeutic drugs in combination with NK600need to be undertaken to confirm the effect of the nanoemulsions and to better understand the underlying mechanisms involved in their anti-cancer activity.

## Figures and Tables

**Table 1:** Protocols in place based on tests performed.

Test	Cell Culture Supports	N° Cells	Experimental Treatments
Viability Assay (MTT)	96 MW	10 × 10^3^/well	1.5–9 µg/mL CCDP Combos: 1.5–9 µg/mL CCDP+ 1.5–9 mg/mL NK600
Proliferation Assay (DNA Assay)	12 MW	80 × 10^3^/well	1.5–9 µg/mL CCDP Combos: 1.5–9 µg/mL CCDP+ 1.5–9 mg/mL NK600
qPCR, WB, GSH	Flask 75 cm^2^	150 × 10^3^/well	6 mg/mL NK600 6 µg/mL CCDP Combo: 6 µg/mL CCDP+ 6 mg/mL NK600

**Table 2:** Genes used for real-time quantitative polymerase chain reaction analysis.

Gene	Assay Type	Amplicon
BCL2L11	SYBR Green	AGACCAAATGGCAAAGCAACCTTCTGATGTAAGTTCTGAGTGTGACCGAGAAGGTA GACAATTGCAGCCTGCGGAGAGGCCTCCCCAGCTCAGACCTGGGGCCCCTACCTCC CTACAGACAGAGCCACAAGGTAATCC
CASP3	SYBR Green	TTCTGAATGTTTCCCTGAGGTTTGCTGCATCGACATCTGTACCAGACCGAGATGTCA TTCCAGTGCTTTTATGAAAATTCTTATTATTAATTATTATACATAAACCCATCTCAGGAT AATCCATTTTATAACTGTTGTCCAGGGATATTCCAGAGTCCATTGATTCGCTTCCAT
GCLC	SYBR Green	GAAGTTATTGTGCAAAGAGCCTGATTTTCTTCTAATATAGAAGTAGCCTCCTTCCGGC GTTTTCGCATGTTGGCCTCAACTGTATTGAACTCGGACATTGTTCCTCCGTAGGGCT GTCCTGGTGTCCCTTCAATCATGTAACTCCCATACTCTGGTCTCCAAAGGGTAGGAT GGTTTGGGTTTGTCCTTTCCCCCTTCTCTTGCAGAGTTTCAAGAACT
GSTP1	SYBR Green	CTCACCCTGTACCAGTCCAATACCATCCTGCGTCACCTGGGCCGCACCCTTGGGCTC TATGGGAAGGACCAGCAGGAGGCAGCCCTGGTGGACATGGTGAATGACGGCGTGG AGGACCTCC
HMOX1	SYBR Green	CATTGCCAGTGCCACCAAGTTCAAGCAGCTCTACCGCTCCCGCATGAACTCCCTGG AGATGACTCCCGCAGTCAGGCAGAGGGTGATAGAAGAGGCCAAGACTGCGTTCCTG CTCAACATCCAGCTCTTTGAGGAGTTGCAGGAGCTGCTGACCCATGACACCAAGGA C
LDOC1	SYBR Green	TGGCATTTTCCAGGTTGGTCCTGATCTCGAAAGGTGGTAGTCTGTAGGTGGGATGTG GTGAGTGGATGTGAAGTGGCAGCATAGTTCTCTGGGAAG
GAPDH	SYBR Green	GTATGACAACGAATTTGGCTACAGCAACAGGGTGGTGGACCTCATGGCCCACATGG CCTCCAAGGAGTAAGACCCCTGGACCACCAGCCCCAGCAAGAGCACAAGAGGAAG AGAGAGACCCTCACTGCTGGGGAGTCCCTGCCACAC

**Table 3:** List of primary antibodies used.

Protein	MW (kDa)	Company
NRF2 (A-10): sc-365949	61	Santa Cruz Biotechnology, Santa Cruz, CA, USA
NF-kB (D14E12)	65	Cell Signaling Technology, Danvers, MA, USA
NF-kB-P (Ser536-93H1)	65	Cell Signaling Technology, Danvers, MA, USA
PARP (46D11)	116/89	Cell Signaling Technology, Danvers, MA, USA
PUMA(D30C10)	23	Cell Signaling Technology, Danvers, MA, USA
P53	53	Cell Signaling Technology, Danvers, MA, USA
HO-1 (TA327035)	32	Origene, Rockville, MD, USA
HDAC1 (H-11): sc-8410	60	Santa Cruz Biotechnology, Santa Cruz, CA, USA
GAPDH (0411): sc-47724	37	Santa Cruz Biotechnology, Santa Cruz, CA, USA

## Data Availability

The data presented in this study are available on request from the corresponding author.

## Supplementary Materials

The following are available online at https://www.mdpi.com/article/10.3390/nano11061365/s1. K600 European Patent Specification.

## References

[1] Siddik Z.H. (2003). Cisplatin: Mode of Cytotoxic Action and Molecular Basis of Resistance. Oncogene.

[2] Borst P., Evers R., Kool M., Wijnholds J. (2000). A Family of Drug Transporters: The Multidrug Resistance-Associated Proteins. J. Natl. Cancer Inst..

[3] Hung C.-C., Chien C.-Y., Chiang W.-F., Lin C.-S., Hour T.-C., Chen H.-R., Wang L.-F., Ko J.-Y., Chang C.-H., Chen J.Y.-F. (2015). P22phox Confers Resistance to Cisplatin, by Blocking Its Entry into the Nucleus. Oncotarget.

[4] Wijdeven R.H., Pang B., Assaraf Y.G., Neefjes J. (2016). Old Drugs, Novel Ways out: Drug Resistance toward Cytotoxic Chemotherapeutics. Drug Resist. Updates..

[5] Omran Z., Scaife P., Stewart S., Rauch C. (2017). Physical and Biological Characteristics of Multi Drug Resistance (MDR): An Integral Approach Considering PH and Drug Resistance in Cancer. Semin. Cancer Biol..

[6] Xu X., Xie K., Zhang X.-Q., Pridgen E.M., Park G.Y., Cui D.S., Shi J., Wu J., Kantoff P.W., Lippard S.J. (2013). Enhancing Tumor Cell Response to Chemotherapy through Nanoparticle-Mediated Codelivery of SiRNA and Cisplatin Prodrug. Proc. Natl. Acad. Sci. USA.

[7] Iyer A.K., Singh A., Ganta S., Amiji M.M. (2013). Role of Integrated Cancer Nanomedicine in Overcoming Drug Resistance. Adv. Drug Deliv. Rev..

[8] Pelicano H., Carney D., Huang P. (2004). ROS Stress in Cancer Cells and Therapeutic Implications. Drug Resist. Updat..

[9] Schumacker P.T. (2006). Reactive Oxygen Species in Cancer Cells: Live by the Sword, Die by the Sword. Cancer Cell.

[10] Trachootham D., Alexandre J., Huang P. (2009). Targeting Cancer Cells by ROS-Mediated Mechanisms: A Radical Therapeutic Approach?. Nat. Rev. Drug Discov..

[11] Trachootham D., Zhou Y., Zhang H., Demizu Y., Chen Z., Pelicano H., Chiao P.J., Achanta G., Arlinghaus R.B., Liu J. (2006). Selective Killing of Oncogenically Transformed Cells through a ROS-Mediated Mechanism by Beta-Phenylethyl Isothiocyanate. Cancer Cell.

[12] Finocchiaro C., Segre O., Fadda M., Monge T., Scigliano M., Schena M., Tinivella M., Tiozzo E., Catalano M.G., Pugliese M. (2012). Effect of N-3 Fatty Acids on Patients with Advanced Lung Cancer: A Double-Blind, Placebo-Controlled Study. Br. J. Nutr..

[13] Miccadei S., Masella R., Mileo A.M., Gessani S. (2016). Ω3 Polyunsaturated Fatty Acids as Immunomodulators in Colorectal Cancer: New Potential Role in Adjuvant Therapies. Front. Immunol..

[14] Sturlan S., Baumgartner M., Roth E., Bachleitner-Hofmann T. (2003). Docosahexaenoic Acid Enhances Arsenic Trioxide-Mediated Apoptosis in Arsenic Trioxide-Resistant HL-60 Cells. Blood.

[15] Baumgartner M., Sturlan S., Roth E., Wessner B., Bachleitner-Hofmann T. (2004). Enhancement of Arsenic Trioxide-Mediated Apoptosis Using Docosahexaenoic Acid in Arsenic Trioxide-Resistant Solid Tumor Cells. Int. J. Cancer.

[16] Pettazzoni P., Pizzimenti S., Toaldo C., Sotomayor P., Tagliavacca L., Liu S., Wang D., Minelli R., Ellis L., Atadja P. (2011). Induction of Cell Cycle Arrest and DNA Damage by the HDAC Inhibitor Panobinostat (LBH589) and the Lipid Peroxidation End Product 4-Hydroxynonenal in Prostate Cancer Cells. Free Radic. Biol. Med..

[17] Kanner J. (2007). Dietary Advanced Lipid Oxidation Endproducts Are Risk Factors to Human Health. Mol. Nutr. Food Res..

[18] Ganta S., Amiji M. (2009). Coadministration of Paclitaxel and Curcumin in Nanoemulsion Formulations To Overcome Multidrug Resistance in Tumor Cells. Mol. Pharm..

[19] Zheng H., Li J., Ning F., Wijaya W., Chen Y., Xiao J., Cao Y., Huang Q. (2021). Improving in Vitro Bioaccessibility and Bioactivity of Carnosic Acid Using a Lecithin-Based Nanoemulsion System. Food Funct..

[20] Desai A., Vyas T., Amiji M. (2008). Cytotoxicity and Apoptosis Enhancement in Brain Tumor Cells Upon Coadministration of Paclitaxel and Ceramide in Nanoemulsion Formulations. J. Pharm. Sci..

[21] Mosmann T. (1983). Rapid Colorimetric Assay for Cellular Growth and Survival: Application to Proliferation and Cytotoxicity Assays. J. Immunol. Methods.

[22] Bijnsdorp I.V., Giovannetti E., Peters G.J., Cree I.A. (2011). Analysis of Drug Interactions. Cancer Cell Culture.

[23] Chou T.-C. (2010). Drug Combination Studies and Their Synergy Quantification Using the Chou-Talalay Method. Cancer Res..

[24] Rao J., Otto W.R. (1992). Fluorimetric DNA Assay for Cell Growth Estimation. Anal. Biochem..

[25] Hissin P.J., Hilf R. (1976). A Fluorometric Method for Determination of Oxidized and Reduced Glutathione in Tissues. Anal. Biochem..

[26] Kaul N., Choi J., Forman H.J. (1998). Transmembrane Redox Signaling Activates NF-ΚB in Macrophages. Free Radic. Biol. Med..

[27] Heo W., Kim J.H., Pan J.H., Kim Y.J. (2016). Lecithin-Based Nano-Emulsification Improves the Bioavailability of Conjugated Linoleic Acid. J. Agric. Food Chem..

[28] Zweers M.L.T., Grijpma D.W., Engbers G.H.M., Feijen J. (2003). The Preparation of Monodisperse Biodegradable Polyester Nanoparticles with a Controlled Size. J. Biomed. Mater. Res..

[29] Yu X., Kensler T. (2005). Nrf2 as a Target for Cancer Chemoprevention. Mutat. Res..

[30] Furfaro A.L., Traverso N., Domenicotti C., Piras S., Moretta L., Marinari U.M., Pronzato M.A., Nitti M. (2016). The Nrf2/HO-1 Axis in Cancer Cell Growth and Chemoresistance. Oxidative Med. Cell. Longev..

[31] Silva M.M., Rocha C.R.R., Kinker G.S., Pelegrini A.L., Menck C.F.M. (2019). The Balance between NRF2/GSH Antioxidant Mediated Pathway and DNA Repair Modulates Cisplatin Resistance in Lung Cancer Cells. Sci. Rep..

[32] Twentyman P., Bagrij T. (1998). The Influence of Glutathione Metabolism on Multidrug Resistance in MRP-Overexpressing Cells. Drug Resist. Updat..

[33] Franklin C.C., Krejsa C.M., Pierce R.H., White C.C., Fausto N., Kavanagh T.J. (2002). Caspase-3-Dependent Cleavage of the Glutamate-l-Cysteine Ligase Catalytic Subunit during Apoptotic Cell Death. Am. J. Pathol..

[34] Traverso N., Ricciarelli R., Nitti M., Marengo B., Furfaro A.L., Pronzato M.A., Marinari U.M., Domenicotti C. (2013). Role of Glutathione in Cancer Progression and Chemoresistance. Oxidative Med. Cell. Longev..

[35] Zheng Z.-G., Xu H., Suo S.-S., Xu X.-L., Ni M.-W., Gu L.-H., Chen W., Wang L.-Y., Zhao Y., Tian B. (2016). The Essential Role of H19 Contributing to Cisplatin Resistance by Regulating Glutathione Metabolism in High-Grade Serous Ovarian Cancer. Sci. Rep..

[36] Brouard S., Otterbein L.E., Anrather J., Tobiasch E., Bach F.H., Choi A.M.K., Soares M.P. (2000). Carbon Monoxide Generated by Heme Oxygenase 1 Suppresses Endothelial Cell Apoptosis. J. Exp. Med..

[37] Lin C.-W., Shen S.-C., Hou W.-C., Yang L.-Y., Chen Y.-C. (2008). Heme Oxygenase-1 Inhibits Breast Cancer Invasion via Suppressing the Expression of Matrix Metalloproteinase-9. Mol. Cancer Ther..

[38] Yee K.S., Vousden K.H. (2005). Complicating the Complexity of P53. Carcinogenesis.

[39] Riley T., Sontag E., Chen P., Levine A. (2008). Transcriptional Control of Human P53-Regulated Genes. Nat. Rev. Mol. Cell Biol..

[40] Nakano K., Vousden K.H. (2001). PUMA, a Novel Proapoptotic Gene, Is Induced by P53. Mol. Cell.

[41] Han J.-w., Flemington C., Houghton A.B., Gu Z., Zambetti G.P., Lutz R.J., Zhu L., Chittenden T. (2001). Expression of Bbc3, a pro-Apoptotic BH3-Only Gene, Is Regulated by Diverse Cell Death and Survival Signals. Proc. Natl. Acad. Sci. USA.

[42] Oda E. (2000). Noxa, a BH3-Only Member of the Bcl-2 Family and Candidate Mediator of P53-Induced Apoptosis. Science.

[43] Yu S.-W. (2002). Mediation of Poly(ADP-Ribose) Polymerase-1-Dependent Cell Death by Apoptosis-Inducing Factor. Science.

[44] Jeffers J.R., Parganas E., Lee Y., Yang C., Wang J., Brennan J., MacLean K.H., Han J., Chittenden T., Ihle J.N. (2003). Puma Is an Essential Mediator of P53-Dependent and -Independent Apoptotic Pathways. Cancer Cell.

[45] Yu J., Zhang L. (2003). No PUMA, No Death. Cancer Cell.

[46] Papaianni E., El Maadidi S., Schejtman A., Neumann S., Maurer U., Marino-Merlo F., Mastino A., Borner C. (2015). Phylogenetically Distant Viruses Use the Same BH3-Only Protein Puma to Trigger Bax/Bak-Dependent Apoptosis of Infected Mouse and Human Cells. PLoS ONE.

[47] Iles N., Rulten S., El-Khamisy S.F., Caldecott K.W. (2007). APLF (C2orf13) Is a Novel Human Protein Involved in the Cellular Response to Chromosomal DNA Strand Breaks. MCB.

[48] Masson M., Niedergang C., Schreiber V., Muller S., Menissier-de Murcia J., de Murcia G. (1998). XRCC1 Is Specifically Associated with Poly(ADP-Ribose) Polymerase and Negatively Regulates Its Activity Following DNA Damage. Mol. Cell. Biol..

[49] Ahel D., Horejsi Z., Wiechens N., Polo S.E., Garcia-Wilson E., Ahel I., Flynn H., Skehel M., West S.C., Jackson S.P. (2009). Poly(ADP-Ribose)-Dependent Regulation of DNA Repair by the Chromatin Remodeling Enzyme ALC1. Science.

[50] Beik J., Khateri M., Khosravi Z., Kamrava S.K., Kooranifar S., Ghaznavi H., Shakeri-Zadeh A. (2019). Gold Nanoparticles in Combinatorial Cancer Therapy Strategies. Coord. Chem. Rev..

[51] Meier J.D., Oliver D.A., Varvares M.A. (2005). Surgical Margin Determination in Head and Neck Oncology: Current Clinical Practice. The Results of an International American Head and Neck Society Member Survey. Head Neck.

[52] Gottesman M.M., Fojo T., Bates S.E. (2002). Multidrug Resistance in Cancer: Role of ATP–Dependent Transporters. Nat. Rev. Cancer.

[53] Peer D., Margalit R. (2006). Fluoxetine and Reversal of Multidrug Resistance. Cancer Lett..

[54] Brown J.M., Wilson W.R. (2004). Exploiting Tumour Hypoxia in Cancer Treatment. Nat. Rev. Cancer.

[55] Moeller B.J., Richardson R.A., Dewhirst M.W. (2007). Hypoxia and Radiotherapy: Opportunities for Improved Outcomes in Cancer Treatment. Cancer Metastasis Rev..

[56] Tian G., Zhang X., Gu Z., Zhao Y. (2015). Recent Advances in Upconversion Nanoparticles-Based Multifunctional Nanocomposites for Combined Cancer Therapy. Adv. Mater..

[57] Lukianova-Hleb E.Y., Ren X., Sawant R.R., Wu X., Torchilin V.P., Lapotko D.O. (2014). On-Demand Intracellular Amplification of Chemoradiation with Cancer-Specific Plasmonic Nanobubbles. Nat. Med..

[58] He C., Lu J., Lin W. (2015). Hybrid Nanoparticles for Combination Therapy of Cancer. J. Control. Release.

[59] Manchado E., Weissmueller S., Morris J.P., Chen C.-C., Wullenkord R., Lujambio A., de Stanchina E., Poirier J.T., Gainor J.F., Corcoran R.B. (2016). A Combinatorial Strategy for Treating KRAS-Mutant Lung Cancer. Nature.

[60] Chen W., Sun Z., Wang X.-J., Jiang T., Huang Z., Fang D., Zhang D.D. (2009). Direct Interaction between Nrf2 and P21Cip1/WAF1 Upregulates the Nrf2-Mediated Antioxidant Response. Mol. Cell.

[61] Asher G. (2005). A Mechanism of Ubiquitin-Independent Proteasomal Degradation of the Tumor Suppressors P53 and P73. Genes Dev..

[62] Kalo E., Kogan-Sakin I., Solomon H., Bar-Nathan E., Shay M., Shetzer Y., Dekel E., Goldfinger N., Buganim Y., Stambolsky P. (2012). Mutant P53 ^R273H^ Attenuates the Expression of Phase 2 Detoxifying Enzymes and Promotes the Survival of Cells with High Levels of Reactive Oxygen Species. J. Cell Sci..

[63] Jiang T., Chen N., Zhao F., Wang X.-J., Kong B., Zheng W., Zhang D.D. (2010). High Levels of Nrf2 Determine Chemoresistance in Type II Endometrial Cancer. Cancer Res..

