# Supplementary material for: Lipoperoxide Nanoemulsion as Adjuvant in Cisplatin Cancer Therapy: In Vitro Study on Human Colon Adenocarcinoma DLD-1 Cells
Source: Nanomaterials (Basel). 2021 May 21;11(6):1365. doi: 10.3390/nano11061365 (PMC8224288; doi:10.3390/nano11061365)
Supplement: Supplementary file 1 [file nanomaterials-11-01365-s001.zip › nanomaterials-1227536-supplementary.pdf]

(19) Europäisches Patentamt
European Patent Office
Office européen des brevets

(11) EP 2 209 470 B1

(12) # EUROPEAN PATENT SPECIFICATION

(45) Date of publication and mention of the grant of the patent:
15.03.2017 Bulletin 2017/11

(21) Application number: **08846226.2**

(22) Date of filing: **06.11.2008**

(51) Int Cl.:
A61K 31/20 (2006.01) ***A61K 31/201*** (2006.01)
A61K 31/202 (2006.01) ***A61K 31/575*** (2006.01)
A61P 1/00 (2006.01) ***A61P 17/00*** (2006.01)
A61P 17/02 (2006.01) ***A61P 27/06*** (2006.01)
A61P 31/04 (2006.01) ***A61P 31/08*** (2006.01)
A61P 31/12 (2006.01) ***A61P 31/18*** (2006.01)
A61P 31/22 (2006.01) ***A61P 35/00*** (2006.01)

(86) International application number:
PCT/IT2008/000693

(87) International publication number:
WO 2009/060493 (14.05.2009 Gazette 2009/20)

(54) **PHARMACEUTICAL COMPOSITION COMPRISING PEROXIDED OIL AND CHOLESTEROL AND USES THEREOF IN MEDICAL FIELD**

ZUSAMMENSETZUNG ENTHALTEND PEROXIDIERTES ÖL UND CHOLESTEROL UND IHRE VERWENDUNG IN DER MEDIZIN

COMPOSITION PHARMACEUTIQUE CONTENANT DE L'HUILE PEROXYDÉE ET DU CHOLESTÉROL, ET SES UTILISATIONS DANS LE DOMAINE MÉDICAL

(84) Designated Contracting States:
AT BE BG CH CY CZ DE DK EE ES FI FR GB GR HR HU IE IS IT LI LT LU LV MC MT NL NO PL PT RO SE SI SK TR

(30) Priority: **08.11.2007 IT RM20070582**

(43) Date of publication of application:
28.07.2010 Bulletin 2010/30

(73) Proprietor: **Carratelli, Mauro**
58100 Grosseto (GR) (IT)

(72) Inventor: **Carratelli, Mauro**
58100 Grosseto (GR) (IT)

(74) Representative: **Gitto, Serena et al**
Barzanò & Zanardo Roma S.p.A.
Via Piemonte, 26
00187 Roma (IT)

(56) References cited:
EP-A- 0 225 831 **EP-A- 0 225 832**
EP-A- 0 481 148 **EP-A- 1 051 979**
EP-A- 1 077 061 **WO-A-2006/102439**
FR-A- 2 591 112 **FR-M- 2 330**

- **SINEGUB G A ET AL: "Treatment of fungal and viral diseases of skin and nails - by applying to affected areas ointment derived from vegetable oil ozonide(s) having peroxide number of 200-700" WPI WORLD PATENT INF,, 1 January 1900 (1900-01-01), XP002941525**
- **HEIMO SCHERZ UND FRIEDRICH SENSER: "Food Composition and Nutrition Tables" 2000, DEUTSCHE FORSCHUNGSANSTALT FÜR LEBENSMITTELCHEMIE, GARCHING B. MÜNCHEN , STUTTGART , XP002520904 p. 173, 179, 181, 188, 189, 192**

Remarks:
The file contains technical information submitted after the application was filed and not included in this specification

EP 2 209 470 B1

Note: Within nine months of the publication of the mention of the grant of the European patent in the European Patent Bulletin, any person may give notice to the European Patent Office of opposition to that patent, in accordance with the Implementing Regulations. Notice of opposition shall not be deemed to have been filed until the opposition fee has been paid. (Art. 99(1) European Patent Convention).

Description

[0001] The present invention concerns a pharmaceutical composition comprising peroxided oil and peroxided cholesterol. Particularly, the present invention concerns a pharmaceutical composition comprising peroxided oil and peroxided cholesterol to be used for the treatment of tumours, viral diseases and ulcers.

[0002] In order to understand the action mechanism of the oil according to the present invention it is worthy to make preliminary considerations. First of all, the cancer cells substantially differentiate in comparison to healthy ones for different energetic metabolism. In fact, it is presently universally accepted that the cancer cells use mainly anaerobic pathway to produce the necessary energy, resulting in the production only of 2 molecules of ATP from one glucose molecule which is transformed in lactate, compared to 36 molecules of ATP produced in healthy cells from one glucose molecule through oxidative phosphorylation using oxygen like combustible. This is the reason for the high avidity of cancer tissue towards glucose (which characteristic allowed diagnostic instruments like the PET to be developed) and the fact that the cancer tissue is strongly acid.

[0003] Secondly, this latter outlined fact results in the breakage of the bond of transition metals with transport and deposition proteins resulting in subsequent release and free state presence thereof in the cytoplasm and microenvironment. Free transition metals can act as catalyst for free radical reactions by reacting with hydroperoxides from blood circulating stream according to the scheme:

$$ROOH + Fe^{++} RO + OH + Fe^{+++}$$

$$ROOH + Fe^{+++} ROO + H^{+} + Fe^{++}$$

[0004] Said radicals are suitable to attack the endothelium vessels which supply the tumour resulting in a cytoarchitecture chaotic and full of little aneurisms. Such situation is particularly damaging for surrounding healthy cells which are deprived of appropriate nutritional supplying.

[0005] Tumour cells in order to be duplicated and construct new membranes need of proteins, lipids and cholesterol.

[0006] The author of present the invention now disclosed that by supplying the cell with peroxided fats according to the invention (fatty acids and cholesterol), the cancer cell dies, because the peroxided fats will find free transition metals and transform in oxygen centered free radicals resulting in cellular apoptosis. Therefore the tumour will liquefy and all the surrounding vessel system will be desiccated. On the contrary healthy cells will be able to use these oxidized fats which will upgrade their metabolism receiving an impulse of hormonal type. It has been moreover found that this stimulus for the cells makes the oil according to the present invention effective for wound healing, treatment of ulcer and dermatitis. However, the stimulus of the cell metabolic activity could represent a risk only when said oxidized fats are administered by systemic route as this can involve an augmentation of the coagulation processes, which, on the other hand, can be controlled using a preventive heparin or anticoagulation therapy. This risk will not occur if the administration will be carried out directly in the cancer area or topically.

[0007] It is therefore an object of the present invention a combination of peroxide olive oil, as needed in association with one or more polyunsaturated oils, for example selected from peroxided soya seed oil, sunflower oil, corn or peanut oil, and peroxide cholesterol, wherein peroxided cholesterol is present at concentration from 1 to 30% of the weight of total fat combination, preferably from 5 to 20%, preferably at concentration corresponding to about 20%. In particular, olive oil and cholesterol, and in the case polyunsaturated oils, are peroxided to values from 100 to 2000 meq/kg of total fat combination, preferably from 800 to 1000 meq/kg.

[0008] The combination according to the present invention can be advantageously employed in medical and veterinary field. In particular, the combination can be used in a separated or sequential way for the therapy of tumours as, for example, thyroid medullary carcinoma, thyroid differentiated carcinoma, mammary carcinoma, colon carcinoma, prostate adenocarcinoma, pulmonary microcytoma, melanoma, glyoblastoma, leukemias.

[0009] Further the combination according to the invention can be employed in a separated or sequential way for the therapy of viral diseases as, for example, Herpes simplex, herpes Zoster, adenovirus, AIDS. In addition the combination according to the present invention can be employed for wound healing, treatment of ulcers, like for example varicose, diabetic, decubitus ulcers.

[0010] Moreover, as a proof that the oil results in a beneficial action on vessel functionality, by exerting anti-edemigen activity, it has been observed that in glaucoma affected patients treated with the oil as eye drops three times daily over a week the intraocular pressure was reduced from two- to threefold resulting in a considerably reduced oedema. This capacity has been also observed in subjects affected by haemorrhoidal oedemas disappearing after 2-3 treatment days. Other very important particularity consisted in that a great benefit has been observed when the oil is used for skin burns, where, if it is applied at a proper time, avoids the lesion occurrence and anyway cellular turn-over is very much accelerated .

[0011] Therefore, the combination according to the present invention can be employed also for the treatment of haemorrhoidal oedemas.

[0012] It is a further object of the present invention a pharmaceutical composition comprising the combination as above defined as an active principle in combination with one or more pharmaceutically acceptable adjuvants and/or excipients.

[0013] The composition or the combination according to the invention can be used in medical and veterinary field. Therefore, the use of the combination and the composition constitutes a further object of the present invention as above defined for the preparation of a medicament for the treatment of tumours, as for example Thyroid Medullary Carcinoma, thyroid differentiated carcinoma, mammary carcinoma, colon carcinoma, prostate adenocarcinoma, pulmonary microcytoma, melanoma, glyoblastoma, leukemias. Further applications of the combination and composition as above defined are for the treatment of viral diseases as for example Herpes simplex, herpes Zoster, adenovirus, AIDS, wound healing, treatment of ulcers, as for example varicose, diabetic and decubitus ulcers, treatment of haemorrhagic and ulcerous colitis, treatment of haemorrhoidal oedemas.

[0014] The combination and the composition according to the present invention can be administered according to the following schedule in effectiveness order:

- Infiltration directed on tumour mass in ml, as 1/10 of tumour mass diameter and small amounts around the mass, repeating the infiltration after 15 days.
- Administration by intravenous systemic route through the suspension of an oil small bottle in an Intralipid 20% 500 ml bag or like, after administration of 4000 low molecular weight heparin units. A bag daily over 10 days. No administration for two days and then 2 cycles, each lasting 10 days with two day interruption.

- Mild therapy: an 8 ml small bottle by oral route, preferably in morning and one 0.4 ml suppository in the evening over three months. Monitor oxidative stress.

[0015] In example 3 cases (namely 2-4 cases) treated with mild therapy (orally administered small bottles and suppositories) are reported.

[0016] The present invention now will be described by an illustrative, but not limitative way according to preferred embodiments thereof.

EXAMPLE 1: *Preparation of peroxided oil according to the present invention*

[0017] Supply possibly BIOLOGICALLY OCCURRING and anyway heavy metal free EXTRAVERGINE OLIVE OIL, heat at about 80°C and dissolve therein certified heavy metal free cholesterol at 10% concentration. If it is desired the percentage to be increased it is suggested to pre-dissolve the cholesterol in high quality polyunsaturated seed oils (for example, soya oil, sunflower oil etc.) and expose this mixture to an UV light source and periodically measure peroxide number till a 500 $mEqO_2/kg$ value of total mixture is reached. The combination according to the present invention can be administered by direct injection, in systemic way through dissolution of the combination in a drip-feed apparatus with lipid suspensions used for parenteral administration, rectally by suppositories, by aerosol route (using nebulizer) after suspension in suitable solvents.

Cytotoxicity

[0018] Tests carried at the Medicina Sperimentale department (DiMeS) of Genoa, in order to estimate the oil cytotoxicity on cell cultures of fibrocytes and endotheliocytes evidenced the oil no-toxicity, indeed demonstrating that the oil increases cellular reproduction, according to what in vivo observed about the acceleration of healing and revascularization processes.

[0019] Tests carried out on tumour cellular cultures have demonstrated instead a significant cytotoxic activity.

EXAMPLE 2: *Effectiveness of the pharmaceutical composition according to the present invention in the treatment of viral diseases*

[0020]

Case 1: 46 year male subject affected by Herpes Simplex treated with the combination of the example 1 simply by touching, 2 times daily. Recovery after 2 days.

Case 2: 80 year female subject affected by Herpes Simplex on a buttock treated by touching in the morning and evening. Recovery after 5 days.

Case 3: 35 year male subject affected by acute painful laryngitis treated by administration using a nebulizer in the morning and evening. Complete full recovery after 2 days

EXAMPLE 3: Effectiveness of the pharmaceutical composition according to the present invention in the treatment of the tumour

[0021]

Case 1: cat with relapsing mammary carcinoma characterised by 2 cm nodule in the sub-mammary tissue. Inoculation of 0.2 ml 800 $mEqO_2$/kg peroxided oil within the nodule and 0.01 ml fractions within surrounding tissue. After 30 days the cat displayed optimal health and the nodule at palpation was very soft. Anyway a sampling of said anatomical portion was carried out for histological assay. Selected nodule was completely liquefied, inside thereof only the involucrum covering fibrotic tissue was present. External tissue vessels appeared to be desiccated. Microscopic analysis displayed only persistence of lymphocytes infiltrations. The cat today displays optimal health.

Case 2: 68 year, colon adenocarcinoma treated, male subject. Relapse after three months. Surgically treated and diffused metastasis observed in all the peritoneum. Again closed and examined by the oncologist for potential chemotherapy. Oil mild treatment started. After a month treatment negativization of Ca 19,9 (from 98 to 32). Optimal clinical conditions, good appetite, optimal deambulation, slightly accelerated evacuation (ampoule resection).

[0022] After one month and 20 days NMR analysis on pelvis results in negative completely outcome. After two months and 15 days execution PET-TAC analysis results in clearly negative results for peritoneum neoformations. Scheduled rectoscopy with potential biopsy. Good general health conditions.

[0023] Case 3: 58 year male subject affected by well differentiated pelvic retro-peritoneal extended through pelvic foramen pelvis lyposarcoma. Disease substantially technically inoperable due to extension. From April mild treatment starts; 8 ml small bottle orally administered in the morning and a 0.4 ml suppository in the evening. Up to now the clinical conditions are good and the last NMR analysis shows liquefaction signs of tumour mass.

[0024] Case 4: 57 year female subject affected by well differentiated, mediastinum extended, pericardium contacting, malignant epithelioid mesothelioma, diagnosis on April 3rd 2008. Due to the extension of the neoformation it is advised against the surgical treatment and the patient, refusing the chemotherapy decides to begin the oral oil ingestion: one 8 ml small bottle in the morning and one suppository in the evening. After five months, the patient displays optimal clinical conditions and the last radiographic assessments would suggest that the neoplastic mass begins to liquefy.

EXAMPLE 4: Effectiveness of the combination according the invention as powerful healing agent for the treatment of any type ulcer or haemorrhoid

[0025]

Case 1: 65 year male subject affected by ulcer due to post-trauma injury: application by touching twice daily. Wound healing after 1 day and then cute strengthening.

Case 2: 60 year haemorrhoid affected male subject resolution after 2 days

Claims

1. Combination of peroxided olive oil, optionally in combination with one or more polyunsaturated oils, and peroxided cholesterol, wherein peroxided cholesterol is present at concentration from 1 to 30% of the weight of total fat combination, preferably from 5 to 20%, preferably at concentration about 20%.

2. Combination according to claim 1, wherein polyunsaturated oils are selected from soya seed oil, sunflower, corn or peanut oil.

3. Combination according to anyone of preceding claims wherein the combination of oil and cholesterol is peroxided to values from 100 to 2000 meq/kg of the total fat combination, preferably from 800 to 1000 meq/kg.

4. Combination according to anyone of preceding claims for use in medical and veterinary field.

5. Combination according to anyone of preceding claims for separated or sequential use in the therapy of tumours.

6. Combination according to claim 5 wherein the tumours are selected from Thyroid Medullary Carcinoma, thyroid differentiated carcinoma, mammary carcinoma, colon carcinoma, prostate adenocarcinoma, pulmonary microcytoma, melanoma, glyoblastoma, leukemias.

7. Combination according to anyone of claims 1-3 for separated or sequential use in the therapy of viral diseases.

8. Combination according to claim 7, wherein the viral diseases are selected from Herpes simplex, herpes Zoster, adenovirus, AIDS.

9. Combination according to anyone of claims 1-3 for separated or sequential use in the treatment of wounds and ulcers.

10. Combination according to claim 9, wherein the ulcers are selected from varicose, diabetic, decubitus ulcers.

11. Combination according to anyone of claims 1-3 for separated or sequential use in the treatment of haemorrhoidal oedemas.

12. Pharmaceutical composition comprising the combination as defined in anyone of claims 1-3 as active principles in combination with one or more pharmaceutically acceptable adjuvants and/or excipients.

13. Pharmaceutical composition according to claim 12 for use in medical and veterinary field.

14. Use of the combination as defined in anyone of claims 1-3 and the pharmaceutical composition as defined in claim 12 for the preparation of a medicament for the treatment of the tumours.

15. Use according to claim 14, wherein the tumours are selected from Thyroid Medullary Carcinoma, thyroid differentiated carcinoma, mammary carcinoma, colon carcinoma, prostate adenocarcinoma, pulmonary microcytoma, melanoma, glyoblastoma, leukemias.

16. Use of the combination as defined in anyone of claims 1-3 and pharmaceutical composition as defined in claim 12 for the preparation of a medicament for treatment of viral diseases.

17. Use according to claim 16, wherein the viral diseases are selected from Herpes simplex, herpes Zoster, adenovirus, AIDS.

18. Use of the combination as defined in anyone of claims 1-3 and pharmaceutical composition as defined in claim 12 for the preparation of a medicament for treatment of wounds and ulcers.

19. Use according to claim 18, wherein ulcers are selected from varicose, diabetic, decubitus ulcers.

20. Use of the combination as defined in anyone of claims 1-3 and pharmaceutical composition as defined in claim 12 for the preparation of a medicament for treatment of haemorrhoidal oedemas.

Patentansprüche

1. Kombination von peroxidiertem Olivenöl, gegebenenfalls in Kombination mit einem oder mehreren mehrfach ungesättigten Öl(en), und peroxidiertem Cholesterin, wobei das peroxidierte Cholesterin in einer Konzentration von 1 bis 30 Gew.-% der gesamten Fettkombination, vorzugsweise von 5 bis 20%, bevorzugter in einer Konzentration von etwa 20%, vorliegt.

2. Kombination nach Anspruch 1, wobei die mehrfach ungesättigten Öle aus Sojaöl, Sonnenblumenöl, Maiskeimöl oder Erdnussöl ausgewählt sind.

3. Kombination nach irgendeinem der vorhergehenden Ansprüche, wobei die Kombination von Öl und Cholesterin auf Werte von 100 bis 2.000 meq/kg der gesamten Fettkombination, vorzugsweise von 800 bis 1.000 meq/kg, peroxidiert ist.

4. Kombination nach irgendeinem der vorgehenden Ansprüche zur Verwendung auf dem Gebiet der Medizin und Veterinärmedizin.

5. Kombination nach irgendeinem der vorhergehenden Ansprüche für die separate oder sequentielle Verwendung bei der Therapie von Tumoren.

6. Kombination nach Anspruch 5, wobei die Tumore aus medullärem Schilddrüsenkarzinom, differenziertem Schilddrüsenkarzinom, Mammakarzinom, Dickdarmkarzinom, Prostataadenokarzinom, Lungenmikrozytom, Melanom, Glioblastom und Leukämien ausgewählt sind.

7. Kombination nach irgendeinem der Ansprüche 1 - 3 für die separate oder sequentielle Verwendung bei der Therapie von viralen Erkrankungen.

8. Kombination nach Anspruch 7, wobei die viralen Erkrankungen aus Herpes simplex, Herpes zoster, Adenovirus und AIDS ausgewählt sind.

9. Kombination nach irgendeinem der Ansprüche 1 - 3 für die separate oder sequentielle Verwendung bei der Behandlung von Wunden und Geschwüren.

10. Kombination nach Anspruch 9, wobei die Geschwüre aus varikösen Geschwüren, diabetischen Geschwüren und Dekubitusgeschwüren ausgewählt sind.

11. Kombination nach irgendeinem der Ansprüche 1- 3 für die separate oder sequentielle Verwendung bei der Behandlung von Hämorrhoidenödemen.

12. Pharmazeutische Zusammensetzung, umfassend die Kombination wie in irgendeinem der Ansprüche 1 - 3 definiert als Wirkstoffe in Kombination mit einem oder mehreren akzeptablen Adjuvantien und/oder Exzipienten.

13. Pharmazeutische Zusammensetzung nach Anspruch 12 zur Verwendung auf dem Gebiet der Medizin und Veterinärmedizin.

14. Verwendung der Kombination wie in irgendeinem der Ansprüche 1 - 3 definiert und der pharmazeutischen Zusammensetzung wie in Anspruch 12 definiert zur Herstellung eines Medikaments für die Behandlung von Tumoren.

15. Verwendung nach Anspruch 14, wobei die Tumore aus medullärem Schilddrüsenkarzinom, differenziertem Schilddrüsenkarzinom, Mammakarzinom, Dickdarmkarzinom, Prostataadenokarzinom, Lungenmikrozytom, Melanom, Glioblastom und Leukämien ausgewählt sind.

16. Verwendung der Kombination wie in irgendeinem der Ansprüche 1 - 3 definiert und der pharmazeutischen Zusammensetzung wie in Anspruch 12 definiert zur Herstellung eines Medikaments für die Behandlung von viralen Erkrankungen.

17. Verwendung nach Anspruch 16, wobei die viralen Erkrankungen aus Herpes simplex, Herpes zoster, Adenovirus, AIDS ausgewählt sind.

18. Verwendung der Kombination wie in irgendeinem der Ansprüche 1 - 3 definiert und der pharmazeutischen Zusammensetzung wie in Anspruch 12 definiert zur Herstellung eines Medikaments für die Behandlung von Wunden und Geschwüren.

19. Verwendung nach Anspruch 18, wobei die Geschwüre aus varikösen Geschwüren, diabetischen Geschwüren und Dekubitusgeschwüren ausgewählt sind.

20. Verwendung der Kombination wie in irgendeinem der Ansprüche 1 - 3 definiert und der pharmazeutischen Zusammensetzung wie in Anspruch 12 definiert zur Herstellung eines Medikaments für die Behandlung von Hämorrhoidenödemen.

Revendications

1. Combinaison d'huile d'olive peroxydée, optionnellement en combinaison avec une ou plusieurs huiles polyinsaturées, et de cholestérol peroxydé, dans laquelle le cholestérol peroxydé est présent en une concentration de 1 à 30% par rapport au poids total de la combinaison de matières grasses, de préférence de 5 à 20%, de préférence en une concentration d'environ 20%.

2. Combinaison selon la revendication 1, dans laquelle les huiles polyinsaturées sont choisies parmi l'huile de graines de soja, huile de tournesol, l'huile de maïs ou l'huile d'arachide.

3. Combinaison selon l'une quelconque des revendications précédentes, dans laquelle la combinaison d'huile et de cholestérol subit une peroxydation jusqu'à des valeurs de 100 à 2 000 méq/kg par rapport au poids total de la combinaison de matières grasses, de préférence de 800 à 1000 méq/kg.

4. Combinaison selon l'une quelconque des revendications précédentes destinée à être utilisée dans le domaine médical et le domaine vétérinaire.

5. Combinaison selon l'une quelconque des revendications précédentes, destinée à être utilisée de manière séparée ou séquentielle dans le traitement de tumeurs.

6. Combinaison selon la revendication 5, dans laquelle les tumeurs sont choisies parmi le carcinome médullaire de la thyroïde, le carcinome différencié de la thyroïde, le carcinome mammaire, le carcinome du côlon, l'adénocarcinome de la prostate, le microcytome pulmonaire, le mélanome, le glyoblastome, les leucémies.

7. Combinaison selon l'une quelconque des revendications 1 à 3 destinée à être utilisée de manière séparée ou séquentielle dans le traitement de maladies virales.

8. Combinaison selon la revendication 7, dans laquelle les maladies virales sont choisies parmi l'herpès simplex, l'herpès Zoster, l'adénovirus et le SIDA.

9. Combinaison selon l'une quelconque des revendications 1 à 3 destinée à être utilisée de manière séparée ou séquentielle dans le traitement de plaies et d'ulcères.

10. Combinaison selon la revendication 9, dans laquelle les ulcères sont choisis parmi les ulcères variqueux, les ulcères diabétiques, les escarres de décubitus.

11. Combinaison selon l'une quelconque des revendications 1 à 3 destinée à être utilisée de manière séparée ou séquentielle dans le traitement d'oedèmes hémorroïdaux.

12. Composition pharmaceutique comprenant la combinaison telle que définie dans l'une quelconque des revendications 1 à 3, en tant que principes actifs en combinaison avec un ou plusieurs adjuvants et/ou excipients acceptables sur le plan pharmaceutique.

13. Composition pharmaceutique selon la revendication 12, destinée à être utilisée dans le domaine médical et le domaine vétérinaire.

14. Utilisation de la combinaison telle que définie dans l'une quelconque des revendications 1 à 3 et de la composition pharmaceutique telle que définie dans la revendication 12 pour la préparation d'un médicament destiné au traitement des tumeurs.

15. Utilisation selon la revendication 14, dans laquelle tumeurs sont choisies parmi le carcinome médullaire de la thyroïde, le carcinome différencié de la thyroïde, le carcinome mammaire, le carcinome du côlon, l'adénocarcinome de la prostate, le microcytome pulmonaire, le mélanome, le glyoblastome, les leucémies.

16. Utilisation de la combinaison telle que définie dans l'une quelconque des revendications 1 à 3 et de la composition pharmaceutique telle que définie dans la revendication 12 pour la préparation d'un médicament destiné au traitement de maladies virales.

17. Utilisation selon la revendication 16, dans laquelle les maladies virales sont choisies parmi l'herpès simplex, l'herpès Zoster, l'adénovirus et le SIDA.

18. Utilisation de la combinaison telle que définie dans l'une quelconque des revendications 1 à 3 et de la composition pharmaceutique telle que définie dans la revendication 12 pour la préparation d'un médicament destiné au traitement de plaies et d'ulcères.

19. Utilisation selon la revendication 18, dans laquelle les ulcères sont choisis parmi les ulcères variqueux, les ulcères diabétiques, les escarres de décubitus.

20. Utilisation de la combinaison telle que définie dans l'une quelconque des revendications 1 à 3 et de la composition pharmaceutique telle que définie dans la revendication 12 pour la préparation d'un médicament destiné au traitement d'oedèmes hémorroïdaux.
